# Community-Based Work and Participatory Budgeting

**DOI:** 10.1177/00027642221086952

**Published:** 2022-04-15

**Authors:** John W. Murphy, Felicia O. Casanova

**Affiliations:** University of Miami, Coral Gables, FL, USA

**Keywords:** Community-based philosophy, Cartesianism/dualism, community interventions, community outreach, community diagnostic, budget assemblies

## Abstract

Participatory budgeting (PB) works best if this activity is viewed to be part of a trend that is referred to as community-based work. But this connection is not often made. As a result, many PB projects tend to drift away from their home communities. Although working in communities is thought to be a very practical endeavor, philosophy should not be ignored, particularly if the aim is to be community-based. Some examples are supplied in this paper that illustrate how this community-based philosophy alters, and improves, some traditional phases of PB projects. The overall result is to keep these budgeting projects informed by local knowledge and under community control.

## Introduction

Participatory budgeting does not exist in a vacuum. This practice is part of trend in social science that is referred to generally as community-based work (CWB). Community-based participatory research, or CBPR, is thought to be the centerpiece of this trend (Israel, et al., 1998). Other examples, however, include citizen science (Bishop, 2014) and popular epidemiology (Brown, 1992). The reason for this sudden interest is quite simple. Advocates of these practices argue that research is improved, along with any policies that are proposed (Abma et al., 2017; Brown et al. 2004; Minkler, 2008). The general result is that the focus of community work has changed, particularly in the area of health (Brown, et al., 2004; Abma, et al. 2017; Minkler, 2008; Israel, 2012).

A serious theoretical gambit made by community-based researchers and practitioners. Simply put, an entirely new philosophy is proposed, along with how research and social interventions should be undertaken (Murphy, 2014). Although at first, community-based work may seem compatible with the qualitative side of research and policy making, this association is tenuous. At the heart of community-based initiatives is a theory of knowledge, or epistemology, that is similar, but a methodological package is advanced that extends beyond a concern for meanings and experiences. In a manner reminiscent of Kuhn (1970), an emerging paradigm is unveiled.

Because this philosophy has been ignored by many persons who engage in CBW, many projects that aim to be community-based never achieve this goal. Participatory budgeting (PB) may fall into this situation. The purpose of this paper, accordingly, is to sketch out some key theoretical issues that must be addressed by anyone who wants to truly engage communities. With some irony, something as practical as designing a community intervention turns out to be a very sophisticated theoretical activity.

Two basic principles guide a community-based project (Murphy, 2014). The first is that local knowledge matters. Every attempt must be made, accordingly, to appreciate how individuals and communities define and navigate their respective realities. The second fundamental theme is that local persons should gradually control all interventions (Geiger, 2002). In other words, the usual role of experts is questioned and eventually assumed by locals. Given that PB involves local persons coming together regularly to discuss and assess issues in their neighborhoods and propose alternative budgets to meet the needs of a particular community, the two axioms that sustain community-based work appear to be relevant.

The point of this discussion is to address systematically a few of the important changes posed by CBW. As is suggested thus far, these changes are not merely technical but involve a change in philosophy that is often overlooked. What this shift in philosophy requires is that a host of seminal themes—some latent and others visible—must be rethought. In other words, a critical eye must be directed at the standard foundations of planning and undertaking social interventions. PB will not likely be successful, for example, if local participation, at the “grassroots” level, is not encouraged. Central to this process is what Orlando Fals Bords (1988) calls “authentic participation,” which extends beyond the usual stakeholder consultations and expert–community partnerships. In other words, community-based projects are guided by local knowledge and controlled by local persons. In these arrangements, local persons become the experts.

### Philosophy and Community-based Work

Local knowledge is central to a community-based project (Kleinman, 1980). The point, however, is not that persons have opinions that deserve attention. Nor is local knowledge a body of convictions, local lore, or sentiments that may affect marginally how behavior or events are perceived. Instead, by appealing to local knowledge supporters of CBW recognize the importance of worlds—individuals and communities enact worlds that specify how all phenomena should be understood (Zaner, 2006). These worlds, however, are not a composite of empirical traits. What is meant by world in this framework refers to the values, beliefs, commitments, and constructions—the existential thread or logic—that situate individuals and communities. This repertoire of information is used daily to differentiate reality from illusion, including the nature of facts and reason. The result is that local realities are envisioned to be an on-going construction and, very likely, different from one another.

For this reason, local control is important (Murphy, 2014). These persons have access to knowledge, at least initially, that is outside of the bailiwick of experts and other outsiders. To borrow from Antonio Gramsci (1971), these persons are organic and thus can provide epistemological entrée to the world that is operative. These individuals, in other words, comprehend the reality that is in play, including the relevant history, cultural themes, language, and so forth. Stated differently, these persons know their local realities and can properly diagnose their communities and prescribe the necessary remedies.

Both local knowledge and control are predicated on participation that jeopardizes a central but latent tenet of traditional interventions. Richard Bernstein (1983) characterized this notion as “Cartesian anxiety.” The idea is that CBW undercuts the Cartesianism that reinforces most social research and community projects. For example, claims about objectivity and evidence-based community service delivery are founded on this dualistic position (Leder, 1984; Sullivan, 1986).

Cartesianism, or dualism, facilitates the categorical separation of the knower from whatever is known; interpretation is thus sequestered from facts (Bordo, 1987). Although operating subtly, this ability to hold interpretation in abeyance is thought traditionally as key to acquiring valid data. Without this prospect, direct encounters with objective facts would be impossible. Any inquiry, and the resulting data or practical initiatives, would thus be muddled by interpretation and anecdotal evidence.

Cartesianism enables society to be portrayed in structural terms so that institutions or other organizations appear to be objective and formidable. On the other hand, this philosophy supports the proposal that subjectivity, and the accompanying meanings, represents opinions and other modes of interpretation that are replete with bias. The local input that is required by PB, accordingly, would be treated as suspect and in need of correction by local professionals, such as budget administrators. After all, participatory budgeting allows local persons, who are mostly untrained, to meddle in the affairs of budget officials. In the past, the only persons who were allowed to participate in this process were trained professionals and a few civil servants, and thus budgeting was portrayed as a rational process. With local involvement, the vision proposed by Cartesianism is violated.

In light of CBW, however, the acquisition of knowledge is fraught with difficulty. The problem is that Cartesianism is subverted due to the inability to overcome participation or human agency. This critique is not exactly new but is a part of 20th-century philosophies such as phenomenology, existentialism, and postmodernism (Bakewell, 2016). Most important, in all of these movements, persons are not blank slates who merely receive and process input. Rather, and crucial for CBW, everyone is constantly interpreting, selecting, and classifying information and thus never simply processing knowledge. Hence, interpretation is everywhere. Knowledge, therefore, is recognized to be replete with imagination and invention. Nothing escapes this condition!

Knowledge, therefore, is never encountered, captured, discovered, or collected. These descriptives are inadequate to depict how interpretation creates knowledge and transforms this information into relevant data. In CBW, data must be coaxed out of the worlds where they reside. This process, accordingly, requires that many standard facets of social planning be revisited and reformulated. These themes must be rethought, specifically, in view of the need to achieve world entry, if valid information is going to be engaged. With dualism passé, a new direction for research and social planning is posed by CBW. Without recognizing this post-Cartesian position, PB will not likely bring about the changes desired by local communities. Budgets will be operationalized as usual, with possibly some citizen consultation near the end of the budgeting process. At this time, the budget calendars of most municipalities provide the opportunity for such involvement.

### Examples of Cartesianism

- The principle mode of dualism present in traditional sociology is the fact–value distinction. The claim, simply put, is that values can be put aside, so that real, reliable knowledge can be uncovered (Hughes & Sharrock, 1997). When the pursuit of knowledge is infiltrated by values, the objectivity of results is thought to be compromised by bias. Without the aid of dualism, values would restrict any claims about accuracy and reliability, thereby undercutting practices or policies. In CBW facts and values are entangled since human agency is never overcome. As a result, facts in community-based projects are treated as local creations rather than objects. In fact, human agency creates a clearing, among competing interpretations, where a particular world is exposed. Those who are taking a community-based tack attempt to engage this creation. What is important at this juncture is that facts are never captured; instead, they should be treated as narratives that must be properly read and interpreted by entering the world that is present.
- The goal of CBW is not to measure anything but to engage a world in dialogue. Without dualism, measurement represents a promise that can never be fulfilled, that is, achieving objectivity and recovering of value-free data (Latour, 1993). As opposed to a disinterested method, the thrust of CBW is to achieve world entrée through dialogue. Throughout most of the history of measurement, the aim has been to capture a particular phenomenon through “accurate, repeatable, numerical” practices (Klein, 1974: 23). What is assumed, however, is that facts are things that can be captured.
  Any community-based project, on the other hand, is thus treated as an interpretive activity, since persons are never exempt from making meaning (Grondin, 1994). Rather than simply revealed or exposed, local knowledge must be enacted or known through participation and proper attunement. Accordingly, the shift that is taking place in CBW is away from measurement to dialogue. Consistent with Gadamer (1989), dialogue is defined as listening to individuals or communities in their own terms, with the assumption that their views can be correctly interpreted. Here is where local knowledge and control become evident in CBW.
- The usual recommendation is that community-based researchers and practitioners should be holistic (De Hoyos, 1989). By this term is meant that they should be encompassing, recognize the complexity of issues, and adopt multiple perspectives on problems, including correctives. In traditional community work, two writers are especially noteworthy, that is, Urie Bronfenbrenner (1994) and James G. Kelly (2006). They are proponents of the ecological model that is Cartesian in character. As a result, holism is conceived abstractly as a system of inter-connected, natural forces that does not resemble daily life. The point should be clear by now that such an ecological whole is not necessarily a world.
  Although CBW may be characterized as primarily a practical affair, by attempting to tinker with local conditions, a dramatic shift in philosophy is underway (Geiger, 2005). With the overcoming of Cartesianism, much of research and social planning should be rethought. The implications of this anti-dualistic maneuver are far reaching and should be taken seriously if the hope is to engage communities. Clearly, interventions should not be foisted on communities, or even undertaken with these persons. These usual distinctions do not go far enough. For example, merely recognizing the need to work with communities does not necessarily address the epistemological shift that is required in CBW, nor the conditions that promote local control.

### Participatory Budgeting and CBW

The PB project that is on-going at the University of Miami has a community-based orientation. The general idea is to train a number of local persons, from underserved communities, in this philosophy and practice. Because this instruction is based on a training-of-trainers model, these initial persons are expected to educate others in their communities to establish participatory budgeting projects. With an expanded network and contacts, a critical mass may be reached to carry this new budgeting process forward and have some local impact.

The project at the University of Miami is couched in community-based philosophy. The curriculum that has been created, accordingly, begins with a discussion of this outlook. Such a strategy is not often followed (Franz & Murphy, 2019). After all, community interventions are typically thought to be very practical affairs, and thus philosophy becomes a distraction. A key point in this special issue, however, is that without this philosophical background, community interventions such as PB will not likely succeed.

As mentioned already, community-based philosophy is anti-Cartesian. In the University of Miami curriculum, this message is conveyed in several ways. At the beginning of the curriculum, the point is stressed that this project represents a new philosophy and that the impact of this change should not be overlooked. In fact, the trainees are told that every facet of planning and implementing a community project, such as PB, must be rethought. They are informed that becoming community-based represents a new philosophy that is tied to practice.

What becomes clear in the training is that the success of PB depends on local participation (Lerner, 2014; Gilman, 2016). In a community-based framework, however, participation is not simply equated with involvement, although joining a community forum and attending budget assemblies are expected. Consistent with an anti-Cartesian stance, the status of knowledge and the portrayal of a community must be reassessed. At the root of apparent practical tasks are some profound philosophical considerations.

Most important is that a fundamental connection is recognized to exist between human action, or agency, and the nature of social life. Stated less abstractly, the values, beliefs, commitments, and interpretations of persons are connected intimately to how knowledge and the identity of a community should be viewed. Personal outlooks and communal visions are affected by how these persons define their situations, including current norms and future expectations.

When assessing life in a community, therefore, interpretation cannot be avoided. Due to the pervasiveness of human agency, striving for objectivity is not an option. To borrow from Max Weber (1978), the best that can be expected is an “adequate interpretation” of behavior and events. The local persons in training at the University of Miami project, therefore, are told to view communities as on-going creations that consist of multiple and often conflicting worlds. Through their daily interaction and solving a myriad of problems, community members accumulate a body of knowledge and a behavioral repertoire that constitute a cultural reality.

Given this challenge to dualism, community work must be approached differently from the past. Cleary, the old-time, top-down strategy that relied on professional expertise is in question, while local participation is elevated in stature. But how will this change affect a community project such as participatory budgeting? While relying on the training offered by the University of Miami project, several examples will be assessed: the community diagnostic, outreach, and the operation of a budget assembly. Each of these stages of a PB project is affected significantly by community-based philosophy.

As noted in the introductory manuscript of this issue, a participatory budget progresses through several stages. Although these phases do not have to be traversed one-after-the-other, there is a logic to their arrangement. Specifically, as a community-based strategy, a PB project should be built from the ground up, with particular attention paid to the influence of human agency.

### Community Diagnostic.

A community diagnostic is carried out early in a PB project (Lerner & Secondo, 2012).

This placement only makes sense since the purpose is to identify what a community desires. Although typically this activity is portrayed as complex, with a myriad of methodological caveats, the aim is not so difficult to achieve. A diagnostic is not designed to specify some fundamental causes of behavior, but to get a general idea of what a community thinks is important. This insight can be obtained by taking a walk about through a community, or by making door-to-door contacts. Even a survey might be used if a community has the resources to conduct such research. Most often, however, very simple procedures are used in community-based projects.

In CBW, in general, the concept of need is skewed because the outcome is mostly negative because underserved communities are judged to have little more than deficits (Kretzmann & McKnight, 1996). The alternative suggestion is that attention should shift to assets—poor communities should be approached like any other and be treated as having both positive and negative traits.

In a community-based project, however, documenting needs or assets may not be sufficient to guarantee that a community is properly understood. A methodological strategy that is often used is social indicator analysis (Land, 1983). According to this method, the physical characteristics of a neighborhood should be the primary focus. The quality of the immediate environment, such as broken windows, cracked side-walks, or the condition of parks, is thought to provide insight into the projects that are appropriate in a community.

The question that is important is, what is evidence? Looking to needs, assets, or social indicators is a Cartesian tactic. That is, all of these phenomena are viewed as objective features that can be surveyed and documented. What is missing from each case is the human element. Evidence is produced through the accumulation of so-called objective data that outline where funds should be redirected through participatory budgeting. Based on this evidence, budget proposals are formulated and prioritized for funding.

So, what is the nature of evidence in a community-based project? Trainees in the University of Miami project are taught that evidence relates to the worlds of communities. Consequently, community elements that seem to be obviously objective are embedded in local narratives and must be properly interpreted. As Paul Ricoeur (1984) notes, the storylines of these worlds are essential to grasping the wants and desires of a community. Nothing should be taken-for-granted with regard to evidence because knowledge accumulation requires an accurate reading of a community.

Because evidence now has a biography, and must be read intently, a diagnostic is not simply a methodological or procedural issue (Berger & Luckmann, 1966). An appropriate reading is not necessarily fostered by more data or increased precision, but rather through dialogue that leads to world entry, and dialogue occurs, according to Gadamer (1989), when local persons are allowed to speak and be heard in their own terms. At this juncture, trainees are instructed that a useful diagnostic requires continuous reflection, an awareness of the interpretive character of facts, and the attempt to understand communities from their perspectives. They must be understood, in short, in terms of how they intend to be understood. A participatory budget proposal will have little relevance if this idea is not appreciated.

### Community Outreach.

Outreach is central to the growth of a PB project. The thrust of this stage is to spread the news about a project; that is, to let people know that participatory budgeting is beginning in a neighborhood (Wampler, 2012). But outreach has other functions, such as spreading the word about meetings and disseminating information about the availability of funds. For example, inviting community members to create neighborhood forums and attend budget assemblies is dependent on competent outreach.

Successfully identifying the so-called target community, however, is essential to productive outreach. As might be expected, Cartesianism comes into play. For example, communities are regularly characterized in an objective manner by researchers and planners (Murphy, 2014). These social arrangements are described as spatial locations, linked to demographic traits, or associated with physical or political boundaries. The Black community or the East End are typical descriptives. The primary task is to circumscribe a community with readily identifiable boundaries. Indeed, political, economic, and other decisions are thought to rest on these dimensions and therefore clarity is sought.

When guided by community-based philosophy, identifying a community is not so simple. All of the objective features that are usually invoked to specify the parameters of a community are subject to interpretation and local (re)negotiation. Where a community begins and ends, in addition to who belongs, are a matter of history, cultural nuances, and commitments. In this sense, physical properties do not stand alone, but are embroiled in interpretations that may not be questioned for a long time. As part of a local narrative, however, a community’s identity can be questioned and edited.

PB could certainly benefit from this rethinking of community. Any portrayal is likely to improve if the emphasis is not placed on irrelevant empirical details. With Cartesianism in doubt, the center of a community is not necessarily a geographic determination. More important is how space is used, that is, where the members of a community walk or congregate (Murphy, et al. 2017). What phenomenologists refer to as the lived space takes precedence over physical dimensions.

How persons, likewise, are associated is not necessarily a physical determination. Propinquity does not lead automatically to communal feelings. In CBW, social solidarity is an outgrowth of discourse and the ties that are generated by this interaction. As persons engage one another, the strength of their associations is specified, despite physical barriers or other spatial determinants. Reaching a community, therefore, should not rely on pinpointing specific buildings or places where fliers or other modes of communication are directed.

Outreach, instead, should be more of an existential undertaking. The aim should be to know where people actually assemble, walk, and seek out information. In a community-based project, these locations are understood to be specified locally, based on how persons interact and identify valid sources of knowledge (Wiesenfeld, 1996). In this sense, discovering the identity of a community may take some effort and time. But this task is not impossible; after all, all of the members seem to know who belongs and who does not, without a map.

The trainees in the University of Miami projects are taught not to mistake spatial and other physical designations for a community. Simply put, a community is not a place but a way of interacting. Outreach, therefore, should be guided by a new sensitivity to how identities are locally constructed and confirmed. And the boundaries of a community are not an exception to this principle. In the end, rather than focusing on formalizing a message, the emphasis of outreach should be on how a community seeks out and interprets news.

### Budget Assemblies.

Budget assemblies are the centerpiece of PB (Mesner, 2018). Here is where proposals are introduced, discussed, and prioritized. PB is brought to fruition in these meetings, as proposals are identified for funding. Preparing to conduct assemblies, therefore, requires skill and attention.

There are many facets of this task, but two are especially noteworthy. The influence of Cartesianism, however, can be found in both. The first pertains to the location of the assemblies. Clearly, these meetings should be accessible to everyone and represent a wide range of persons. A proper location is critical to fostering adequate attendance.

When influenced by Cartesianism, the solution to this issue is quite straightforward. A central location is thought to be the proper place. Most often, however, this placement is assumed to be synonymous with the geographic center that is equidistant from everyone. But this version of space and location may have nothing to do with life in a community. As noted above, perception and local judgment are everything when attempting to navigate a community. In this regard, how a community is demarcated is a local decision that should be respected. What some persons believe is far away or near, for example, may be interpreted very differently in various communities.

The second consideration relates to the organization of an assembly; Cartesianism may be lurking in the organization of these meetings. The point is that all attendees should be encouraged to speak and their ideas given a fair hearing. How seating is arranged may influence whether persons feel comfortable talking out loud. Likewise, there are several technical procedures available, such as the Delbecq method, to prevent some persons from monopolizing a discussion (Delbecq et al., 1975). The aim of these practices is to promote a sense of inclusivity.

There is another factor, indicative of Cartesianism, that may work against achieving this end. Specifically, a discussion may be restricted because essentialism may pervade an assembly (Fish, 1989). This factor is operative, for example, when organizers presume that good ideas are likely to emerge only from select parts of a community. What participants say from a particular segment may be judged more harshly or dismissed altogether because of the traits associated with these persons. Are particular persons or groups thought to be more insightful or motivated than others?

Those who engage in PB, however, may view themselves to be progressive and overlook this discrimination. Nonetheless, this problem has become quite worrisome, given the prevalence of implicit bias exhibited by many types of groups (Smedley et al., 2003). Given this latent source of bias, essentialism may go unchallenged and begin to shape budget assemblies. But attributing inherent traits to persons without the assistance of Cartesianism is difficult because identities are not natural but interpersonally or culturally constituted. In light of community-based philosophy, vigilance is maintained to root out such attribution.

The outcomes of assemblies are expected to be as inclusive as possible. Therefore, the location and organization of these meetings play a vital part in guaranteeing that funding decisions represent and uplift an entire community. When those choices are inclusive, no one is left behind and identified as losers. In the absence of Cartesian-inspired essentialism, a democratic culture can be created in these meetings where everyone belongs and proposals proliferate.

## Conclusion

Participatory budgeting is part of a new imagination, whereby a new moral economy can be established. But clearly, this change does not occur overnight. As neighbors begin to interact regularly and learn from one another, true solidarity may begin to form (Su, 2016). As they begin to exhibit concern for one another, trust may emerge. With this sort of cohesion, and the accompanying mutual aid, a community may begin to act and pursue goals together.

The basic hope is that persons will no longer act in a self-interested manner but will forge a communal presence. When this change happens, the usual centers of action, or power, may begin to shift. As local capacities develop, the agendas and spheres of influence of communities may also expand. To use a term popularized by social psychologists, these persons may acquire a more potent sense of efficacy (Bandura, 1997). In other words, they may begin to feel that they have some control of their futures.

But in community-based work, the term empowerment is a misnomer (Rappaport, 1987). Through PB, communities do not suddenly gain power; they have always had the ability to improve their lives and situations. What is illustrated through PB, instead, are some of the processes that have marginalized certain communities and kept them from flourishing. In this particular case, the underside of municipal budgeting can be revealed and correctives proposed that may improve communities, particularly those that have been traditionally underserved.

PB is not a silver bullet that cures everything in a community. Redirecting funds away from the police toward health, for example, must be accompanied by serious community organization and political will. This new budgeting process, nonetheless, can help to build local skills, promote a sense of civic responsibility, and create the vision that communities can act in unison to deal with societal problems (Gilman, 2016). In this sense, a more expansive role for citizens may be revealed and internalized. In the end, PB serves not merely as a means to rearrange budget priorities, but to encourage the growth of a more participatory and democratic culture.
